# Cultural-Responsiveness of the Mental Health First Aid Training for Asian Immigrant Populations in Greater Boston, Massachusetts

**DOI:** 10.21203/rs.3.rs-3449868/v1

**Published:** 2023-12-02

**Authors:** Min Kyung Kim, Grace S. Su, Angel N.Y. Chan, Yuxin Fu, Yanqing Huang, Chien-Chi Huang, Ben Hires, MyDzung Chu

**Affiliations:** Tufts Clinical and Translational Science Institute; Boston Chinatown Neighborhood Center (BCNC); Boston Chinatown Neighborhood Center (BCNC); Boston Chinatown Neighborhood Center (BCNC); Institute for Clinical Research and Health Policy Studies, Tufts Medical Center; Asian Women for Health (AWFH); Boston Chinatown Neighborhood Center (BCNC); Institute for Clinical Research and Health Policy Studies, Tufts Medical Center

**Keywords:** Asian mental health, mental health training, Mental Health First Aid, community based participatory research, cultural responsiveness, cultural appropriateness, Greater Boston

## Abstract

**Background:**

The COVID-19 pandemic and rise in anti-Asian racism have had adverse mental health impacts in Asian communities. The lack of culturally-responsive and linguistically-accessible mental health trainings hinders access to mental health services for Asian populations. In this study, we assessed the mental health needs of Asian communities in Greater Boston and evaluated cultural responsiveness of the Mental Health First Aid (MHFA), a first-responder training teaching participants skills to recognize signs of mental health and substance use challenges, and how to appropriately respond.

**Methods:**

This community-based participatory research with the Boston Chinatown Neighborhood Center (BCNC), Asian Women For Health (AWFH), and the Addressing Disparities in Asian Populations through Translational Research (ADAPT) Coalition employed two phases. In phase 1, we conducted focus groups with BCNC and AWFH staff and peer educators to assess mental health priorities of Asian populations in Boston. Findings informed phase 2, which evaluated cultural responsiveness of the MHFA through pre- and post-training questionnaires and focus groups with community participants. The pre-training questionnaire asked about mental health needs and barriers, help-seeking behaviors, and literacy; and personal and Asian community stigma. The post-training questionnaire and focus group with community participants asked about cultural competence of MHFA training for Asian populations. Paired t-tests were used to evaluate questionnaire responses. Thematic analysis was used to analyze interviews.

**Results:**

In total, 10 staff/educators and 8 community members participated in focus groups. They identified common mental health needs and workforce and culturally-responsive community strategies to support persons with mental health issues. Twenty-four community participants completed pre- and post-training questionnaires. They reported the MHFA training reduced mental health care stigma and increased mental health literacy. Recommendations to increase cultural-responsiveness of the MHFA were to include mental health case studies common in Asian populations and provide the training in other languages (e.g., Chinese, Vietnamese).

**Conclusion:**

Cultural responsiveness of the MHFA for Asian populations could be improved with the inclusion of case studies specific to the Asian communities and accessibility of the training in other languages. Increasing the cultural relevance and language accessibility of these trainings could help reduce mental health stigma and gaps in mental health awareness and service utilization among Asian populations.

## Background

The COVID-19 pandemic compounded with the rise in anti-Asian racism have had significant mental health impacts in Asian communities in the US, with more than 4 in 10 Asian Americans reporting current mental health symptoms in 2021(1). In Greater Boston, mental health continues to be a top community health concern (2). In 2021, approximately 22% of Asian students in Boston seriously contemplated suicide (3). Recent studies also highlight increasing financial insecurity, workplace health and safety concerns, crowding, racial discrimination (4), and problem gambling (5) in Asian communities throughout Greater Boston. These mental health risk factors are associated with increased symptoms of anxiety and depression, diminished life satisfaction, and suicidal ideation (5, 6). Yet, Asians have the lowest rates of mental health service utilization (7, 8), with significant barriers to access such as the lack of culturally-responsive and linguistically-accessible providers, long wait lists for care, and stigmatization (2, 9). In addition, the model minority myth, which portrays Asians as a monolithic and universally healthy group, reinforces poor data collection which leads to limited funding and mental health services dedicated to Asian communities (10, 11).

One approach to increasing the availability of culturally-responsive mental health service is through engagement with community-based organizations (CBOs). CBOs serve as cultural liaisons in underserved communities and provide essential, linguistically and culturally-appropriate care to clients whose needs are often unmet in mainstream services (5, 12). Throughout the pandemic, CBOs have had to rapidly respond to increasing demands for mental health support services among their clients (4, 13, 14). For instance, the Orange County Asian Pacific Islander Community Alliance’s staff and their community health workers provided mental health workshops to support Asian students and families through virtual education sessions and mental health telehealth services during the COVID-19 pandemic (15, 16). However, the lack of funding and workforce with culturally competent mental health training has hindered CBO’s capacity to sufficiently respond to the growing demand for mental health services. In addition, most existing community-level trainings are not culturally-tailored or linguistically accessible to Asian populations.

A widely recognized community-level mental health training is the Mental Health First Aid (MHFA). The MHFA is a mental health first-responder program that teaches community members to recognize risk factors and warning signs for poor mental health and substance use concerns, and strategies to appropriately help someone in both crisis and non-crisis situations (17). The MHFA training has been implemented in several Asian immigrant communities globally (e.g., Chinese and Vietnamese) and has shown to improve mental health literacy and stigma (18–20). Among a Chinese community in Australia, the MHFA training improved the level of mental health literacy and reduced stigmatizing attitudes (18). However, it has not been implemented in Asian immigrant communities in the U.S. and is currently only available in English and Spanish.

To better understand whether the MHFA training can be utilized as community-based training for Asian populations in Greater Boston, MA, we evaluated the utility and cultural responsiveness of the MHFA training to address mental health conditions and care needs for Asian populations in Greater Boston. We also conducted focus groups with staff and peer educators from two local CBOs to better understand the context of mental health among Asians in Greater Boston.

## Methods

### Study design

This study employed a community-based participatory research (CBPR) approach (21) in collaboration with the ADAPT (Addressing Disparities in Asian Populations through Translational Research Coalition) at Tufts Clinical Translational Science Institute (CTSI)) Coalition and two CBOs: the Boston Chinatown Neighborhood Center (BCNC) and Asian Women for Health (AWFH). ADAPT is a community-academic partnership based in Boston’s Chinatown (22), of which BCNC and AWFH have been core community partners since 2011. BCNC provides a range of family-centered programs to approximately 13,000 children, youth, and adults from Greater Boston including Chinatown, Quincy, and Malden, MA each year (13). AWFH is a peer-led, community-based network dedicated to advancing Asian women’s health and wellbeing across 11 partner sites in Boston’s Chinatown, Somerville, Dorchester, Roxbury, and Worcester (14). The objectives of this study are aligned with BCNC and AWFH’s programmatic goals to address community mental health needs in the Asian community. This study received the Institutional Review Board (IRB) approval from Tufts Medical Center/Tufts University (reference number: 00003155).

### MHFA training

The MHFA training program is an evidence-based curriculum informed by guidelines developed using the Delphi consensus method, which involves the formation of an expert panel on what constitutes a best practice MHFA (23). The training teaches participants risk factors, warning signs, and symptoms of various mental illness (e.g., depression, anxiety, trauma, psychosis, substance use disorders, self-injury, and suicidal behaviors). The synchronous instructor-led component includes interactive learning activities such as case-based learning, role play, and self-reflection. In addition, participants learn a structured response, e.g., ALGEE, consisting of five actions:

1. **A**ssess risk of suicide or harm
2. **L**isten non-judgmentally
3. **G**ive reassurance and information
4. **E**ncourage the person to get appropriate professional help
5. **E**ncourage self-help strategies

MHFA training provides two courses: the *Youth MHFA* training that teaches how to help adolescents (age 12–18) experiencing mental health challenges (24), and the *Adult MHFA* training that teaches how to help adults (age 18+) (25). The MHFA training program can be delivered in three formats: 1) in-person, 2) blended, and 3) online. Each format lasts about 8 hours, with the blended format involving 2 hours of self-paced online learning and 6 hours in-person training. Participants are also provided a MHFA course manual. The MHFA program has more than 750 instructors delivering trainings globally, and organizations in 14 countries have adopted the program (18). More information about the MHFA has been previously described (26).

### Delivery of the training

In 2022, two staff from BCNC and AWFH received the Youth and Adult MHFA instructor trainings, respectively. After being qualified as MHFA instructors, BCNC facilitated three Youth MHFA trainings via blended and online formats from December 2022 to May 2023 with 41 participants. AWFH facilitated two adult MHFA trainings via an online format in January 2023 with 8 participants.

### Recruitment of study populations

The study population included two target groups:

#### 1) BCNC staff and AWFH staff/peer educators for focus group discussions.

Eligibility criteria for participation were 1) at least 18 years old and 2) work/interact with Asian populations in the areas of mental health. In total, two virtual focus groups, one for each organization, were facilitated. Each focus group included five staff and lasted approximately 60 minutes via a secured Zoom link approved by the study IRB. After the discussion, each participant received $20 electronic gift cards for their time and participation. In total, 10 BCNC and AWFH staff/peer educators participated in the focus group.

#### 2) Community participants who completed the MHFA training for structured questionnaire and focus group discussions.

Eligibility criteria were 1) anyone affiliated with the Asian communities in the Greater Boston area, 2) at least 18 years old, and 3) enrolled in either BCNC or AWFH’s MHFA training. Recruitment for community participants occurred after they registered for the MHFA training. Participation in the research study was voluntary and separate from MHFA training. Participants were asked to complete 1) an online survey (about 10-minutes long) before (pre-) and after (post-) the MHFA training, and 2) a virtual focus group discussion (about 60 minutes). Once the participant completed the MHFA training, the study RA sent the invitation for focus group discussions. If participants completed the pre- and post-MHFA questionnaires, they received a $15 electronic gift card. Those who participated in the focus groups received an additional $15 electronic gift card in compensation for their time and participation. In total, 8 community participants participated in the focus group.

### Data collection

#### Focus Group Discussion

We facilitated focus group discussions with BCNC and AWFH staff and peer educators to learn more about mental health conditions and care needs for Asian populations in the Greater Boston area. We asked five open-ended questions about Asian mental health needs; common mental health seeking behaviors; training and education needed to support Asian community members with mental illnesses; and cultural, community, familial, language, and immigrant-related contexts to consider when working with Asian community members.

We also facilitated focus group discussions with community participants who completed the MHFA to learn more about the utility and cultural responsiveness of the MHFA training. We asked five open-ended questions about common mental health conditions and challenges among Asian peers; the most and least relevant parts of the MHFA training for Asian populations; specific Asian communities that can benefit from the training; and any recommendations for MHFA trainings to be culturally responsive for Asian populations.

#### Pre- and Post-training questionnaires

The pre-MHFA online questionnaire was sent to participants one week prior to their MHFA training date. The questionnaire consisted of 69 questions across seven sections (see **Supplemental file 1**), comprising of the following:

1. Demographics (e.g., race/ethnicity, nativity, age), involvement with Asian-serving CBOs, and experience participating in the MHFA in the past.
2. Self-reported health, asked “In general, would you say your health is?’ with five response options - excellent, very good, good, fair, and poor.
3. Potential barriers to mental health care based on the Barriers to Access to Care Evaluation (BACE) Scale (27). Participants were asked, “Whether an Asian person who had mental health problem ever stopped, delayed, or were discouraged from getting or continuing with professional care for a mental health problem?” based on 15 barriers (e.g., lack of health insurance) using a four-point Likert response scale (0 = not at all to 4 = a lot)
4. Perception of the likelihood of mental health help-seeking behaviors for Asian persons with mental health issues based on a list of 12 resources/services (e.g., intimate partner, traditional healer) using a three-point Likert scale response (0 = Not likely to 2 = Very likely)
5. Availability of linguistically-accessible and culturally-responsive mental health services in the Asian community (e.g. “Is this service provide in language accessible to Asian non-English speakers?”; “Does this service have culturally-competent providers who have worked with Asian patients before?”) with binary response options (yes/no) based on help-seeking questionnaire (28, 29).
6. Personal and community-level stigma to address mental health issues based on a vignette of a case of depression (30, 31). The same vignette was used for participants in the adult and youth MHFA trainings, but the subject’s age varied (e.g., a 30-year old adult and vs. a 15-year old teenager, respectively). To measure personal stigma, respondents were asked, “*Please indicate how strongly YOU PERSONALLY agree or disagree with each statement*,” by rating 9 statements (e.g., people with problems like Kim could snap out of it if they wanted) using a five-point Likert scale (1 = strongly disagree to 5 = strongly agree). To measure Asian community stigma, respondents were asked to respond from the perspective of the Asian and Asian American communities (“*Please indicate how MOST PEOPLE in the ASIAN/ASIAN AMERICAN COMMUNITY agree or disagree with each statement*”, to each of the 9 statements (e.g.., most people in the Asian community believe that people with problems like Kim could snap out of it if they wanted) using a five-point Likert scale. Scores were summed and averaged across 9 statements, with low scores reflecting less stigmatizing attitudes.
7. General mental health literacy (30) and mental health literacy specific to Asian populations (30, 32–35). The participants were asked to rate whether six knowledge-based statements on mental health issues in the general population (e.g., *Around half of mental disorders starts during childhood* or adolescent) and six statements on mental health issues specific to Asian populations (e.g., S*uicide is the leading cause of death for Asian youth ages 15–24 years old*) were True or False. Responses were scored as follows: correct (+ 1), incorrect (−1) and don’t know (0). Scores were summed such that higher scores reflect higher literacy on mental health. The maximum possible score was 12.

Immediately following the completion of MHFA training, participants were sent the post-MHFA online questionnaire, which consisted of 32 questions (see **Supplemental file 2**). The three sections on demographics, self-reported health, and Asian community stigma from the pre-questionnaire were excluded. Also, for the section on potential barriers to mental health care, the question was modified in the post-training questionnaire to ask whether common potential barriers were covered in the MHFA training (e.g., “Did the MHFA training that you just completed help you recognized any of the following potential barriers to individuals seeking mental health care? (Select all that apply)”) from a list of 19 barriers (e.g., lack of insurance coverage, concern that people may find out, fear of medication side effects). The post-training questionnaire also asked participants for free-text feedback about cultural competence of MHFA training to Asian and immigrant populations (e.g., During the MHFA training, were there any specific examples or case studies that were tailored to Asian populations?).

### Statistical analyses

#### Qualitative analysis

Thematic analysis in five phases was used to analyze the focus group data (36). The first phase involved two reviewers familiarizing themselves with the data through repeated reading and transcription. The second phase involved two reviewers independently generating initial codes of the entire data. During the third phase, two reviewers searched for themes via reviewing, defining, and naming themes. During the fourth phase, all themes were reviewed by discussing the relationship among the themes, presenting them in a more systematic way as a map/figure, and refined themes (e.g., merging subthemes that have overlapping meanings). In case of differences in coding, two reviewers discussed themes and made a consensus on one theme and/or include as a subtheme. Phase five involved writing and preparing a manuscript, including selecting quotes that represent the essence of each theme.

#### Quantitative analyses

Descriptive statistics of the study population were assessed. Mean and standard deviation of personal and Asian community stigma and mental health literacy were calculated. Paired t-test was used to compare personal versus Asian community stigma from the pre-training questionnaire, and changes in personal stigma, mental health literacy from pre-training to post-training. Percent response rate was calculated for 1) literacy on the needs and barriers to mental health care self-reported by participants, 2) mental health help-seeking behavior for Asian members, and 3) availability of mental health services in Asian communities. All analyses were conducted in Stata SE v17.0 (37) and statistical significance was set at an alpha-level of 0.05 for all analyses.

## Results

In total, 24 community participants (BCNC: n = 21, AWFH: n = 3) completed pre- and post-training questionnaires (Table 1). The majority (91%) of participants identified themselves as Asian, non-Hispanic, and primarily of Chinese ethnicity (Table 1). About half of participants were foreign-born (50%) and 72% of participants had at least a Bachelor’s degree.

### Context of mental health needs in the Asian community in Greater Boston

The level of community-level stigma against mental health help-seeking behaviors perceived by participants from the Asian community (mean 3.84, Standard Deviation [SD] 0.51) was more than twice the level of self-reported personal-level stigma (mean 1.73, SD 0.70) (p < 0.001) (Table 2).

The most common barriers to *seeking* care identified by participants were stigma from family members (88%), concern that people might find out (79%), inability to describe or express one’s mental health issues (79%), and dislike of talking about one’s feelings, emotions, or thoughts (79%). In addition, participants reported structural barriers to *accessing* care, such as the lack of insurance coverage (74%) and financial costs (67%) (Table 3).

**Figure 1:**
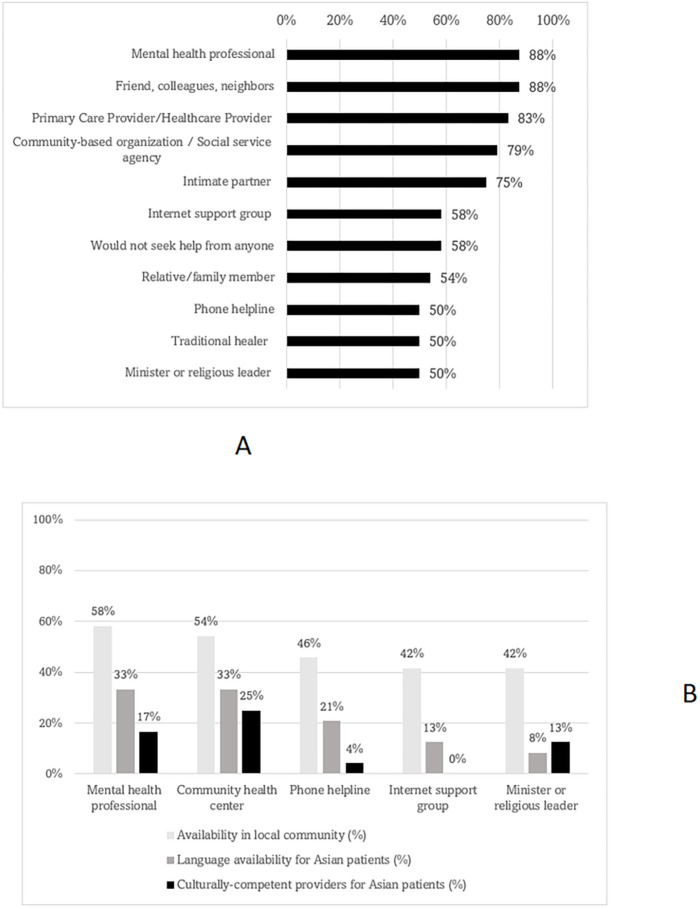
a – Likelihood of mental health being sought from the following persons or outlets (N=24) b – Availability of mental health services/supports in local community (N=24)

The majority (88%) of participants identified both formal (e.g., mental health professionals) and informal (e.g., friend, colleagues, neighbors) resources as outlets to seek mental health support for Asian community members (Fig. 1a). However, the availability of mental health services and supports in the local community was perceived to be low (mental health professional: 58%; community health center: 54%). In addition, access to in-language (33%) and culturally-competent providers (17%) for Asian, non-English speaking patients was also perceived to be low in the local community (Fig. 1b).

### Focus group with CBO staff and peer educators

**Figure 2:**
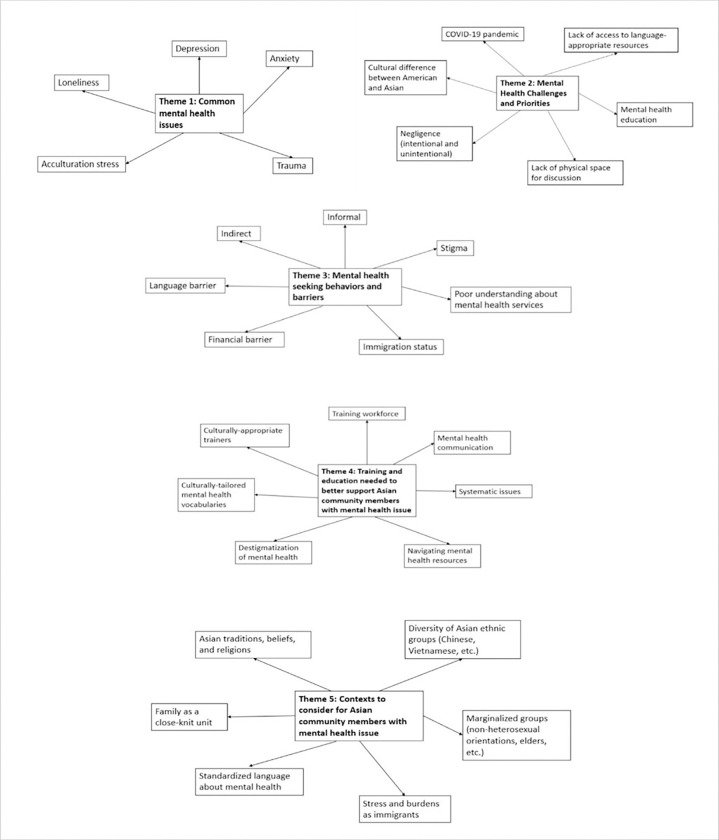
Thematic mapping from focus group with staff (N=10)

During the focus group with CBO staff and peer educators, five major themes were discussed (Fig. 2, **Supplemental table 3**). For common mental health issues (Theme 1), participants identified the COVID-19 pandemic as a major cause of the increase in mental health conditions and challenges among Asian community members in recent years:

“We’re hearing a lot about isolation, feeling lonely, among mostly elders as well as older adults who are kind of homebound during COVID.”

“The anti-Asian hate crimes for the past couple of years, a lot of them [e.g. Asian residents] are just terrified.”

Other common mental health issues were loneliness, depression, anxiety (e.g., social, financial difficulty), trauma (e.g., immigration, generational), and acculturation stress:

People whose parents were immigrants have that survival mentality, and I think that feeds into their children having a lot of anxiety, depression, and being unable to navigate the world.

Staff/peer educators also identified mental health challenges and priorities (Theme 2) such as the lack of access to language-appropriate resources; American versus Asian cultural differences in gender expectations and parent-child communication; lack of personal space and conflicting environments between home and school:

“Even the topic of talking about mental health and talking about therapy is also difficult, too, because they [parents] don’t fully understand what that means or how to make their children feel loved and supported.”

Other challenges raised were intentional negligence due to long wait times (e.g.., deferring mental health care for other priorities), unintentional negligence (e.g., unawareness of mental distress), limited mental health education, and lack of physical recreational spaces to relieve mental distress:

You know [Asian Americans adults] are not going to go to a bar or have drinks with their friends. They want to go to karaoke or playing ping pong or something like that

With respect to mental health help-seeking behaviors in the Asian community (Theme 3), staff/peer educators shared that many of their clients used indirect approaches (e.g., engagement in family services or afterschool programs) and informal methods (e.g., online search, friends, religious leaders) to seek care options:

“It’s very rare, at least based on my anecdotal experience, that clients come specifically to seek mental health assistance. It’s usually something that comes up in the discussion about other things [like family service, afterschool program]”

In terms of mental health seeking barriers, staff/peer educators frequently mentioned stigma (e.g., worry about other members finding out), and the model minority myth:

“One thing that’s unique in the Asian community is that we put less emphasis on individual than the fact we treat family as a unit, so there’s really not much privacy per say”

“…mental health needs and distress are easily brushed off as a flaw of character”

Other common barriers included poor understanding about mental health services (e.g., limited language about mental health resources, mental health vocabularies, lack of trust in mental health system), financial barriers (e.g., high cost, prioritizing basic needs first), and language barriers (e.g., lack of Asian language speaking counselors and bilingual translators), and immigration status (e.g., documentation):

I’ve heard that a lot of undocumented folks are really afraid of seeking service, and when they are seeking services, it’s still hard for the provider to really build trust quickly, because [undocumented folks are] kind of afraid that things they get into or the services they seek will get them into trouble and I think that’s another thing to keep in mind.

The staff/peer educators also pointed out a great need for culturally-responsive mental health workforce training, communication strategies, and education (Theme 4) to better support Asian community members with mental health issues:

“A lot of the trainings I received I feel like were built for predominantly White culture”

It’s important to have some type of training or education for the community about systemic issues in America, the injustices that’s happening to kind of break the myth, like the model minority myth Types of communication strategies included more appropriate description of mental distresses and intergenerational dialogue between parents and children.

Key contexts to consider when addressing mental health issues in the Asian community (Theme 5) included: Asian traditional, beliefs, religions, medical practice; variations among Asian ethnic groups (e.g., Chinese vs. Vietnamese), and the incongruence with standardized language (e.g., needing glossary for translation, lost in translation).

Different ethnic groups go to different treatment centers or community health centers.

“I’ve had someone who actually told me about how her lungs are having too much heat. In [Asian] medicine, I think that’s relating to anxiety.”

Other important contexts raised were the close-knit nature of the family units and community that could contribute mistrust, lack of privacy, and high judgment; the stress and burdens that immigrants experience (e.g., traumatic, outsider/foreigner treatment), and within culture marginalization of specific groups:

The invisible judgment I get asked quite often like, is my neighbor going to see me talking to you?

“[Immigrants] not having the life they used to have just because they moved to a different country, it’s sort of something that’s an ongoing struggle for them.”

“For a lot of gay and trans folks that are within the Asian community, they might face getting kicked out [of their homes].”

### Focus group with community participants

**Figure 3:**
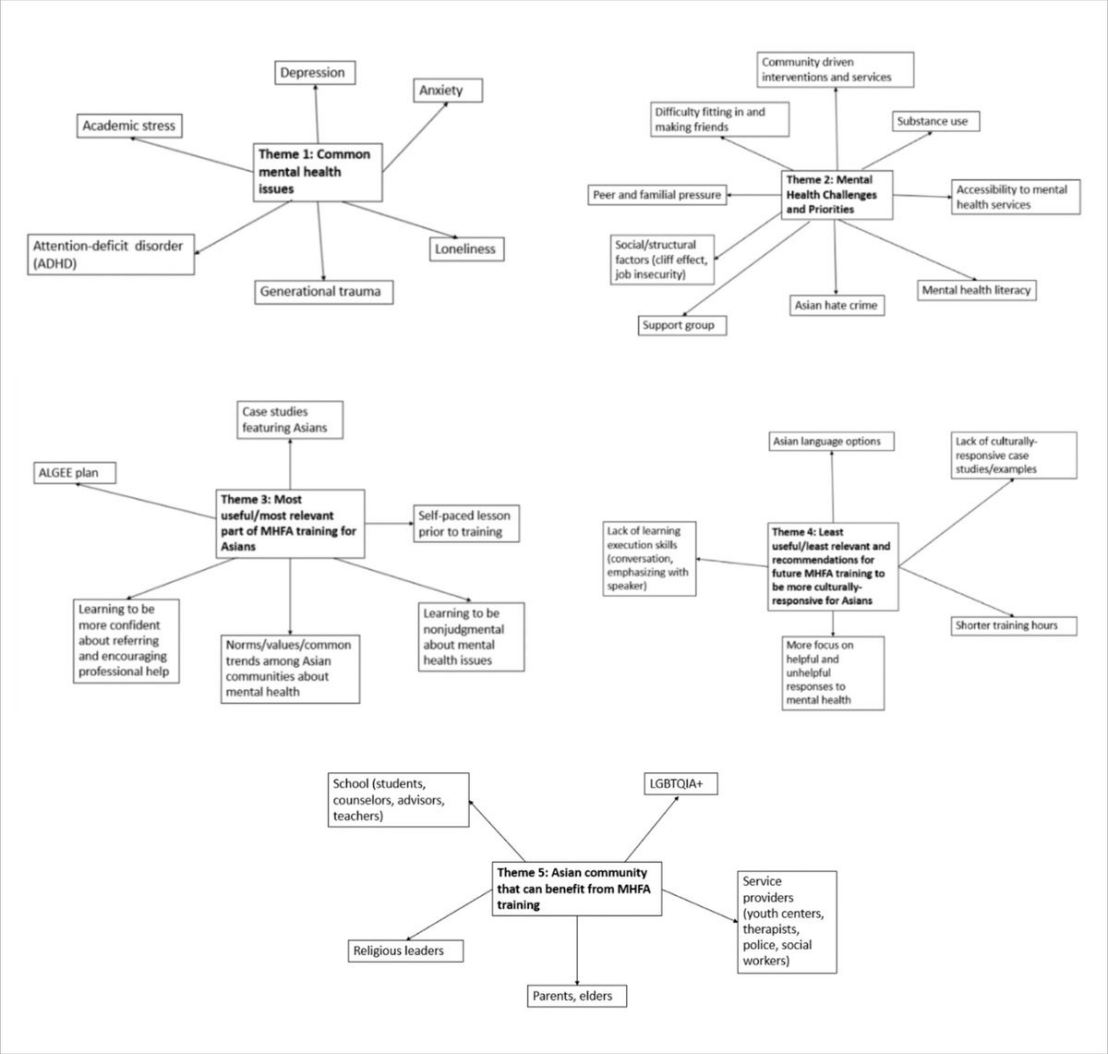
Thematic mapping from focus group with community participants (N=8)

During the focus group with community participants (Fig. 3, **Supplemental table 4**), academic stress, depression, anxiety, loneliness, attention-deficit/hyperactivity disorder (ADHD), and generational trauma were identified as common mental health issues among their Asian peers:

Very often I feel that parents are projecting their life values, such as becoming a doctor or becoming a lawyer or getting married, and these projecting of life values can have a huge toll on the children.

Community participants identified the following mental health challenges and priorities for the Asian community: difficulty fitting in and making friends in America, substance use (e.g., marijuana), peer and familial pressures, and difficulty confiding personal issues to “strangers” (e.g., therapists). The increase in Asian hate crimes during COVID-19, limited accessibility to mental health services, poor mental health literacy, and loss of safety nets (e.g., cliff effect in public benefits, job insecurity, housing insecurity) were also important social and structural factors impeding access to mental health services:

“Every time we make a referral, they will say, ‘Oh, the waiting is at least for six months or even more than a year,’ so it is very challenging for them to get services right away”

“A lot of times, it’s framed black and white, where you’re either in crisis or not, and if you’re not, you don’t need help”

“We’re kind of stuck on being poor all the time in order to be on MassHealth so we can pay, we can get medical coverage, our medication, see our therapists, our doctors.”

Community participants also shared about the value of support and skills-based groups for residents to “come together and discuss self-help resources”, as well as community-driven mental health interventions and services.

### Utility and Cultural-responsiveness of the MHFA

Almost all (92%) participants said they would recommend the MHFA training to another community member (data not shown). Personal stigma to address mental health issues significantly decreased from pre-training (mean 1.73, SD 0.70) to post-training (mean 1.38, SD 0.44) (p < 0.001). Also, participants’ mental health literacy improved from pre-training (mean 5.96, SD 2.44) to post-training (mean 7.17, SD 2.94) (p = 0.04) (Table 2).

In focus groups with community participants (Fig. 3, **Supplemental table 4**), the most useful and relevant parts of the MHFA training for Asians were the ALGEE plan, self-paced lessons prior to training, the case study featuring an Asian family reported by few participants, and discussions about norms/values/common trends among Asian communities about mental health (e.g., high expectations/high functioning).

Learning about the ALGEE plan helps me give background knowledge on the things I can do if I were in the situation of providing mental health support.

After completing the MHFA training, community participants felt more non-judgmental towards individuals experiencing mental health issues (e.g., taking time to understand, not pushing them to make decisions). Participants also reported feeling more confident about referring and encouraging professional help among Asians (Fig. 3, **Supplemental table 4**).

Barriers to mental health care pertaining to stigma and mental health literacy were reported to be covered in the MHFA training by community participants. However, barriers related to personal issues, such as unwillingness to improve (21%) and preference to only seek help from family and friends (46%), and the lack of insurance coverage (58%) were not covered in the MHFA, despite most participants identifying them as a concern in the Asian community (Table 3).

Moreover, 62% of community participants reported that there were no examples or case studies tailored to Asian populations, while a third recalled only 1 or 2 examples or case studies. Similarly, the majority of participants (86%) reported no examples or case studies tailored to immigrant and refugee populations (**Supplemental table 5a**).

In both the focus groups (Fig. 3, **Supplemental table 4**) and post-training questionnaires (**Supplemental tables 5a & 5b**), community participants identified several limitations in the cultural-responsiveness of the MHFA for Asian and immigrant populations: the lack of learning execution skills (e.g., conversation, emphasizing with speaker); limited case studies/examples specific to the Asian and immigrant/refugee communities (e.g., immigrant status, family dynamic, academic stress); and the lack of language options.

Participants suggested following areas in which the MHFA to be improved on to be more culturally-responsive to mental health issues in the Asian communities:

“I thought it would be useful to talk more about the stigma and how to have those conversations with different generations or people with very different views”

“I think definitely the case study scenario discussion can include more cultural awareness, for example, based on new immigrants or undocumented immigrants.”

“…more on like dealing with academic stress and intergenerational struggle.”

“…getting more context as to why folks might be resistant to seeking help in regard to Asian cultures. I think more stress on not placing the blame on the individual links to that.”

Discuss the concepts of shame, respect, and filial piety embedded in Asian culture and how they affect Asians at different stages in life.

Participants also shared specific feedback to improve the culturally-responsiveness of the MHFA to address to mental health issues in the immigrant and refugee communities:

“Learning more about the history of those populations and why it may affect mental health.”

“Show specific cases about immigrant/refugee individuals.”

Community participants recommended that the MHFA training be provided in other Asian languages (e.g., Chinese-Mandarin, Chinese-Cantonese, Korean, Japanese, and Vietnamese). Other general considerations for future MHFA trainings included shorter training hours (e.g., workshop series, multiple days), more focus on helpful and unhelpful responses to mental health (**Supplemental table 4**), expanding more on what it means to be culturally sensitive; and more coverage of topics such as: *how to provide psychoeducation about seeking professional help; how to deal with a person who refuses help; parent-child communication role playing* (**Supplemental tables 5a & 5b**). Community participants also suggested the following sectors and community stakeholders who would also benefit from taking the MHFA trainings: school staff (e.g., counselors, teachers), service providers (e.g., youth centers, police, social workers), the LGBTQIA + community, parents and elders, and religious leaders (Fig. 3, **Supplemental table 4**).

## Discussion

Since the COVID-19 pandemic, there has been a notable increase in public health awareness about mental health as a critical priority in Asian communities (1, 9). However, systemic deficiencies in the number of culturally-responsive and linguistically-accessible mental health providers for Asian populations have created a growing chasm between demand and supply (9, 11). As one potential alternative to narrow this gap, the MHFA training could be an accessible and timely way to train community members with the skills and recognition to identify risk factors of poor mental health and appropriately intervene.

This study evaluated mental health needs of the Asian community in the Greater Boston area and the utility and cultural responsiveness of MHFA training for Asian populations, particularly Chinese immigrants. We found that stigma against mental health care was perceived to be higher in the Asian community than at the individual level. Lack of culturally responsive and linguistically-accessible mental health training, workforce, and community strategies were identified by community organizations and community participants as ongoing causes of poor utilization of mental health services in the Asian community. The MHFA training improved mental health literacy and reduced stigma related to mental health care among Asian participants. However, the lack of culturally relevant case studies in the MHFA training and limited language options beyond English and Spanish to take the training hindered its cultural responsiveness and linguistic accessibility to Asian populations, many of whom are immigrants and non-English speakers.

Cultural, geographic, and immigration-specific contexts are important when addressing mental health care needs in Asian communities. Our findings in Greater Boston, particularly for Chinese residents, suggest that the current MHFA may be poorly adapted to the diverse mental health challenges of Asian, immigrant, and limited English-speaking populations in the U.S. While the MHFA training has been implemented in a few Asian communities outside the U.S. (18, 19, 38) and has demonstrated improvements in participants’ recognition of mental disorders, only one study has been conducted in the U.S. This study looked at the MHFA’s cultural relevance to Bhutanese refugee community (19) and found no reduction in negative attitudes towards people with mental illness. In addition, both CBO staff and community members shared that the emphasis on filial piety in most collectivist Asian cultures often lead to academic stress and intergenerational struggles, which pose mental health challenges that are distinct from those arising from the more individualistic American culture. Notably, a quarter of Asians in the U.S. (27%) live in multigenerational households (39).

In addition, language access was consistently named as a priority for CBOs and community participants in the Asian communities. Asian populations in the U.S. represent over 19 different ethnic groups and 10 different languages (40). Asians also comprise the largest foreign-born populations in the U.S. (41), with only 57% being English-proficient (39). Language inaccessibility is a major barrier to MHFA participation as well as the capacity of trainers to apply their skills in limited English-speaking and immigrant Asian communities. Currently, only English and Spanish language versions of the MHFA trainings are available in the U.S. (17). However, in the next few years, the National Council for Mental Wellbeing that oversees the MHFA in the U.S. plans to translate its youth and adult MHFA curriculum to other languages such as Chinese, Korean, and Khmer (ref: internal communications), which marks an important step towards increasing the MHFA’s diversity, inclusion, and mental health equity for diverse Asian populations.

Furthermore, despite a reduction of stigma against mental health care observed among Asian participants in the MHFA training, community participants and staff/peer educators from local CBOs still identified stigma as the major reason for poor mental health service utilization in Asian communities. Our findings revealed that perceived mental health stigma in the Asian community was two-folds higher than personal stigma, reinforcing the need to address stigma at the community-level. In 2021, Asians showed the lowest utilization rate of mental health services (25%) compared to other racial/ethnic groups (36%–52%) in the U.S. (42). Compounding this issue, the model minority myth perpetuates the misconception that Asians are inherently healthier than other racialized minority groups (10, 16). Given that stigma within the Asian communities surpass personal stigma levels, providing MHFA trainings directly to community members by collaborating with CBOs could be an effective way to address deeply-rooted stigma within the Asian communities and foster a more supportive environment that encourages help-seeking and the receipt of mental health support (16, 43).

### Recommendations and Future Directions

To address these unique challenges, it is important that the MHFA and similar mental health training resources assess and improve their cultural-responsiveness and linguistic-accessibility to better align with the diverse and unique mental health needs and experiences in the Asian communities. These resources should be made available in other languages predominant in these communities, such as Chinese (Cantonese and Mandarin), Hindi, Tagalog, and Vietnamese (39). In addition, these resources should include health statistics and case examples that are specific to Asian populations as well as representative of the diverse ethnic groups and their cultural, geographic, and immigration-related contexts. For example, Asian high school youth in the U.S. (29%) showed a higher rate of suicidal thoughts and behaviors than the overall U.S. youth population (19%). Poverty rates in the U.S. vary greatly across Asian ethnic groups, ranging from 6% among Indians to 25% among Mongolian and Burmese groups (40). In addition, discrimination against Asians and anti-Asian hate crimes have also substantially increased during the COVID-19 pandemic (44). Culturally- and population-specific case examples could be added in the MHFA curriculum to help trainees better contextualize and address these alarming mental health inequities for Asian community members.

### Strengths and limitations

This study is one of the first to evaluate the MHFA training among Asian community participants in the U.S. We used a CBPR approach in collaboration with local CBOs that promoted community empowerment, capacity-building, and co-learning (45). Additionally, this study used a mixed-methods approach to assess mental health needs and evaluate whether the MHFA addressed these needs specific to the Asian community.

Our study also has several limitations. First, the evaluation of the MHFA training included non-randomized group assignment. We used a convenience sampling method to increase survey uptake and recruited CBO staff/peer educators and community participants from two local organizations that primarily served Chinese immigrant populations. This approach likely limited the representation of diverse Asian ethnic groups as well as the geographic representation to urban populations. Second, while our evaluation demonstrated a reduction in personal stigma against mental health care and an increase in mental health literacy among participants, it is difficult to know whether these improvements were sustained over time. Our evaluation period was short-term, with the pre- and post-questionnaire administered within a week of the MHFA training. Future studies should consider including more long-term follow-up.

## Conclusion

The MHFA training was found to be an effective tool at reducing personal stigmatizing attitudes towards people with mental illness and improving knowledge about mental disorders and appropriate response strategies to support persons in crises within the Asian community in the Greater Boston area. However, the cultural-responsiveness of the MHFA training for Asian populations can be improved by including more health statistics and case studies specific to these populations. Specifically, differences in cultural, geographic, and immigration-related contexts across diverse Asian ethnicities need to be considered. In addition, more language options for the MHFA will increase its access and use among Asian and immigrant communities which often have the lowest mental health service utilization despite the high prevalence of mental health challenges. Given that stigma against mental health care is higher at the Asian community level than individual level, collaborating with CBOs to offer trainings at the community level could be an effective strategy to increase the utilization of mental health services.

## Figures and Tables

**Table 1 –:** Demographics of Community Participants for Pre- and Post-Training Surveys (N = 24)

	N (%)
**Number of community participants**	
Adult MHFA– AWFH^a^ sponsored	3 (13%)
Youth MHFA – BCNC^b^ sponsored	21 (87%)
**Gender**^c^ (self-identified)	
Female	16 (76%)
Another gender (i.e., male, non-binary, agender)	5 (24%)
**Age**^c^ (range, in years)	22–66
**Race/Ethnicity** (self-identified)	
Chinese, non-Hispanic	16 (67%)
Another Asian, non-Hispanic (e.g., Filipino/Filipina, non-specify Asian)	6 (25%)
Another race/ethnicity (e.g., White, Black)	2 (8%)
**Nativity**	
US-born	12 (46%)
Foreign-born	11 (50%)
Missing	1 (4%)
**Self-reported health**	
Excellent	2 (8%)
Very good	13 (54%)
Good	7 (29%)
Fair	2 (8%)
**Education** ^c^	
High school and/or Associate degree	6 (29%)
Bachelor’s degree	6 (29%)
Graduate degree	9 (43%)

<5 indicates the number of responses less than 5. Community-based organizations:

a Asian Women For Health (AWFH)

b Boston Chinatown Neighborhood Center.

c Data for gender, age, and education level were only available for 21 participants who completed BCNC training.

**Table 2 –:** Comparison of Mental Health Literacy and Stigma. Pre- and Post-Training Surveys (N = 24)

	Pre-training scores	Post-training scores	p-value
	Mean (SD)	Mean (SD)	
**Stigma** ^a^			
Personal	1.73 (0.70)	1.38 (0.44)	*< 0.001* ^b^
Asian community	3.84 (0.51)	Not asked	NA
	*< 0.0001* ^c^	NA	
**Mental Health Literacy** ^d^	5.96 (2.44)	7.17 (2.94)	*0.04* ^e^

a Stigma for personal and Asian community was assessed using case vignettes using scoring matrix: strongly agreed (5), agree (4), neither agree or disagree (3), disagree (2), and strongly disagree (1). Higher score indicates higher stigma perception. Standard deviation (SD)

b Paired t-test of pre- and post-training surveys were used to calculate the p-value for the Personal Stigma category. P< 0.05 is considered statistically significant.

c Paired t-test of pre-training responses for the Personal vs. Asian community categories was used to calculate this p-value. P < 0.05 is considered statistically significant.

d Mental health literacy was based on 12 statements about mental health in general population and Asian population with scoring matrix: correct (+1), incorrect (−1), don’t know (0). Higher score indicates higher literacy about mental health. Maximum possible score was 12.

e Paired t-test of pre- and post-training surveys were used to calculate the p-value for the Mental Health Literacy category. P< 0.05 is considered statistically significant.

**Table 3 –:** Literacy about Potential Barriers to Mental Health Care and Coverage in the MHFA trainings (N = 24)

	Identified by participants before the training^a^	Topics covered by MHFA training^b^
**Stigma**		
Concern about stigma from family member(s)	88%	88%
Feeling embarrassed or ashamed by community member(s)	71%	83%
Concern that people might find out	79%	83%
**Mental health literacy**		
Lack of knowledge on where to get the professional care	67%	83%
Lack of professionals from individual’s own ethnic or cultural group	63%	67%
Inability to describe or express one’s mental health issues	79%	67%
**Personal issues**		
Slow progress of mental health care	71%	50%
Preference to only seek help from family or friends	71%	46%
Unwillingness to improve	63%	21%
Preference to only seek alternative forms of help	54%	33%
Dislike of talking about one’s feelings, emotions or thoughts	79%	88%
**Social determinants of health**		
Lack of insurance coverage	74%	58%
Financial costs	67%	75%
Lack of transportation	54%	46%

a This is self-reported by participants completed on pre-training survey, this question was coded as whether each potential barrier to mental health care ever stopped, delayed, or discouraged a person who identifies as an Asian/Asian-American and has or has had a mental health problem in a range of: This has stopped Not AT ALL (0), Don’t Know (0), This has stopped A LITTLE (1), QUITE A LOT (1), AND A LOT (1).

b This is self-reported by participants completed on post-training survey, this question was coded as whether the Mental Health First Aid training help participant from recognizing any of the following potential barriers by select all that apply. If the respond checked the question, we coded as one (1). If the box was not checked, we coded as zero (0).

## Data Availability

All the data supporting our findings have been presented in the manuscript; the datasets used and/or analyzed during the current study are available from the corresponding author on reasonable request.

## Abbreviations

CBO: community-based organization
MHFA: Mental Health First Aid
CBPR: community-based participatory research
BCNC: Boston Chinatown Neighborhood Center
AWFH: Asian Women for Health
IRB: Institutional Review Board
ALGEE: **A**ssess risk of suicide or harm, **L**isten non-judgmentally, **G**ive reassurance and information, **E**ncourage the person to get appropriate professional help, **E**ncourage self-help strategies
ADAPT: Addressing Disparities in Asian Populations through Translational Research
CTSI: Clinical Translational Science Institute
